# Supplementary material for: The Supported Boro-Additive Effect for the Selective Recovery of Dy Elements from Rare-Earth-Elements-Based Magnets
Source: Materials (Basel). 2022 Apr 21;15(9):3032. doi: 10.3390/ma15093032 (PMC9101444; doi:10.3390/ma15093032)
Supplement: Supplementary file 1 [file materials-15-03032-s001.zip › materials-1680180-supplementary.pdf]

Supplementary

FeB alloy has to be homogeneous since existence of any other phases can disturb to observe reaction behavior following by phase stability. Quantitative analysis and phase analysis of FeB alloy are observed to confirm composition and uniformity of phase.

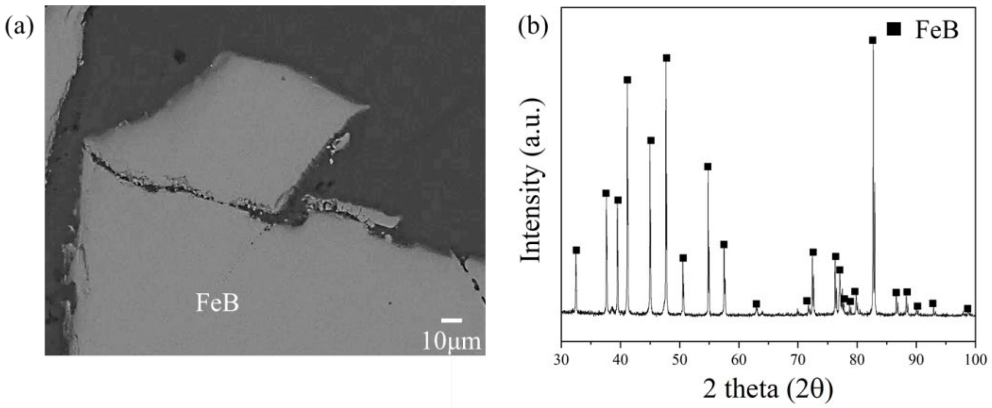

Figure S1. (a) Microstructure and (b) phase confirmation of input FeB alloy

Table S1. Composition of input FeB.

Composition (wt.%)	Fe	B	Minor Elements
FeB	88.58	16.22	0.2

To prevent the growth of Fe_2B layer, the amount of FeB is adjusted below 0.07 which is expected composition. Figure 2 (b) and (c) show the microstructure of reduced the composition to half and one third, respectively. While the specimen of FeB alloy as half shows the growth Fe_2B layers and REE-borides still remained, the reacted zone is dominant without the Fe_2B layer in the other case.

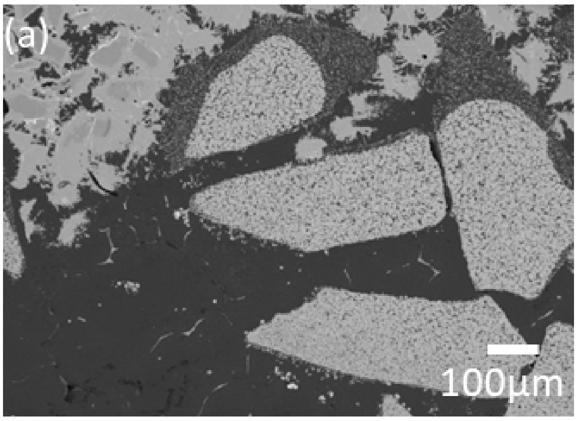

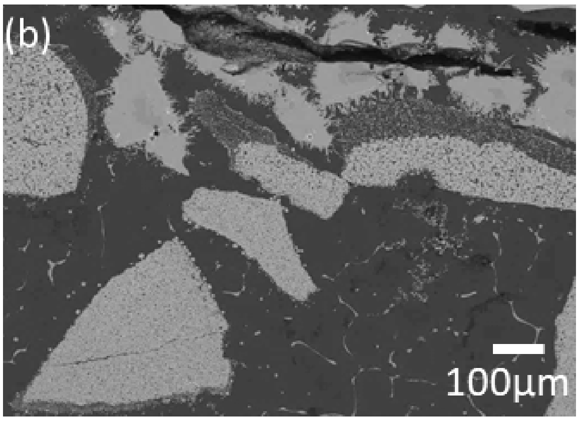

Figure S2. Microstructures after FeB reduced reaction experiments as (a) half and (b) one third
